# A Rare Case of Job Syndrome With Autism: Complicated With Hidradenitis Suppurativa and Chronic Deep Vein Thrombosis

**DOI:** 10.7759/cureus.20832

**Published:** 2021-12-30

**Authors:** Tutul Chowdhury, Malavika Shankar, Nicole Gousy, Bilal Siddique

**Affiliations:** 1 Internal Medicine, One Brooklyn Health System, Brooklyn, USA; 2 Medicine, American University of Antigua, New York, USA; 3 Biochemistry, Avicenna Medical College, Lahore, PAK

**Keywords:** hyper immunoglobulin e syndrome, chronic deep vein thrombosis, autism spectrum disorder (asd), hidradenitis suppurativa complication, job’s syndrome

## Abstract

Job syndrome or hyper-immunoglobulin E syndrome is one of the rare immunologic diseases with only about 300 cases described in the literature until now. Given their low prevalence, our understanding of both autosomal dominant and recessive Job syndromes is still evolving. No specific treatment options are available but early diagnosis may help in treating cases prophylactically with antibiotics and wound care to reduce the patient’s burden. We recently encountered a patient diagnosed with Job syndrome with autism who presented with an abscess in the right axillary region. We report this case for its rarity and unique association with developmental neurologic disorder. It is crucial to review this rare syndrome to circumvent any diagnostic delay. Following the disease course and taking all the associations into account is also vital for the clinician’s understanding as well as implementing the treatment plan.

## Introduction

Hyper-immunoglobulin E syndrome (HIES), which is also known as Job syndrome, was first described in 1966 in two girls, although it does not have any gender or ethnic predominance [1,2]. HIES is a rare immune disorder with both autosomal dominant and recessive inheritance linked to STAT3 gene mutation resulting in defect of Th17 cell genesis and modulation capacity of interleukin 6 and 10 [3-6]. STAT3 gene is widely involved in modulating healing and the immune mechanism. This rare primary immune deficiency manifests with recurrent skin and lung infection, dermatitis, and high IgE count [1,2]. Opportunistic infections like *Pneumocystis jiroveci* pneumonia and disseminated histoplasmosis are not uncommon [4]. Musculoskeletal systems also can be involved with varying degrees of scoliosis, minimal trauma fractures, degenerative joint disease, and osteopenia [3,4]. Features like craniosynostosis, hyperextensibility of the joints, multiple bone fractures, and coarse facial features have also been pointed, which are not linked with the immune system [6,7]. Primary immunodeficiencies are considered to be associated with an increased incidence of malignancy but are not evidenced yet in the case of Job syndrome and need to be studied exponentially [8].

## Case presentation

**Figure 1:**
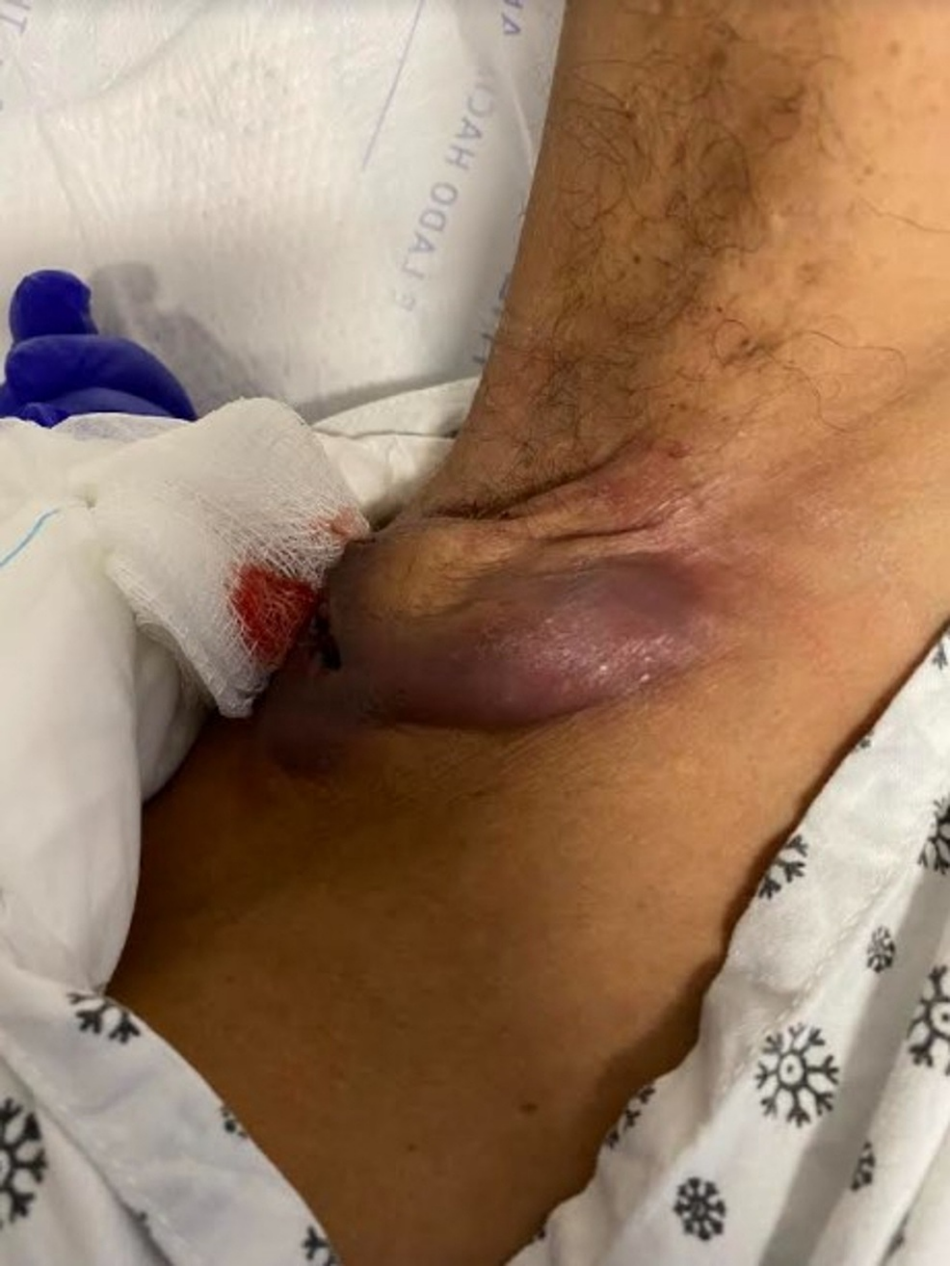
The right axillary abscess that the patient presented with.

**Figure 2:**
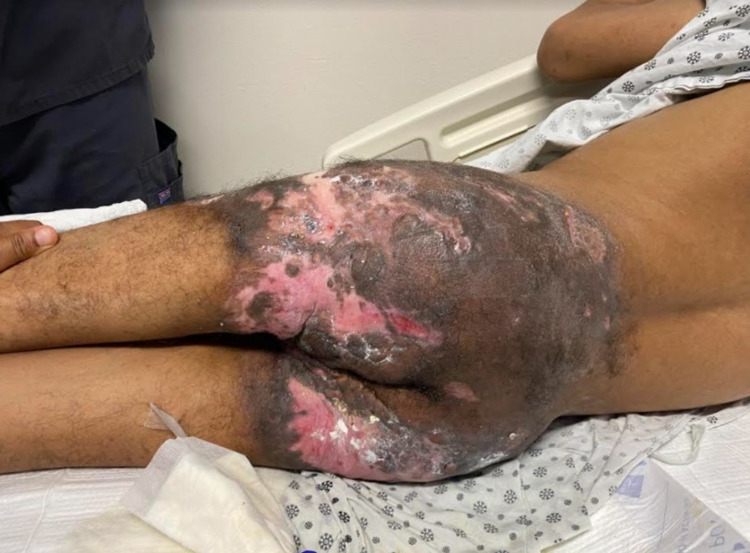
The diffuse sacral and gluteal hyper-pigmentation, ulceration, and scarring due to the patient's history of recurrent abscesses with concurrent immobility of his lower extremities.

Our patient is a 35-year-old male, a diagnosed case of hyper-IgE syndrome with a past medical history significant for autism spectrum disorder, anemia, prior pulmonary embolism, multiple gluteal abscesses, and bilateral lower limb paralysis secondary to trauma at 15 years of age. This patient presented with a right axilla abscess and limited range of motion of the right arm noticed by the patient’s mother for three days (Figure 1). The patient is alert and awake but non-verbal at baseline, so history was obtained from the mother at the bedside and previous hospital admission records. The patient started having numerous recurring abscesses starting at 8 years old, leading to a diagnosis of hyper IgE syndrome at age 18. Microbiology of the abscesses over the last year have grown *Pseudomonas aeruginosa*, Group C Streptococcus, and beta-hemolytic Streptococci that were susceptible to penicillin and other beta-lactams and treated with such. Due to his history of recurrent abscesses, there are large patches, most notably in the sacral and gluteal area, of scarring, hyper-pigmentation, and ulceration (Figure 2).

Clinical examination showed a nonverbal adult male alert and oriented in moderate distress due to severe right arm pain. Consistent with his prior diagnosis of the hyper-IgE syndrome, this patient had coarse facial features and significant scoliosis. Vital signs were hemodynamically stable, and the patient was afebrile despite having a large purulent abscess in the right axilla. Additionally, there were multiple patches of erythematous rashes and skin infections visible, with healed sinus tracks noticed around the groin and scrotal area. Swelling of the right axilla with visible abscess drainage severely limits the patient’s range of motion of the right arm due to pain. Given his immunocompromised state due to hyper-IgE syndrome, the patient was admitted for the administration of broad-spectrum antibiotics including vancomycin, cefepime, and doxycycline until culture could be obtained.

**Figure 3:**
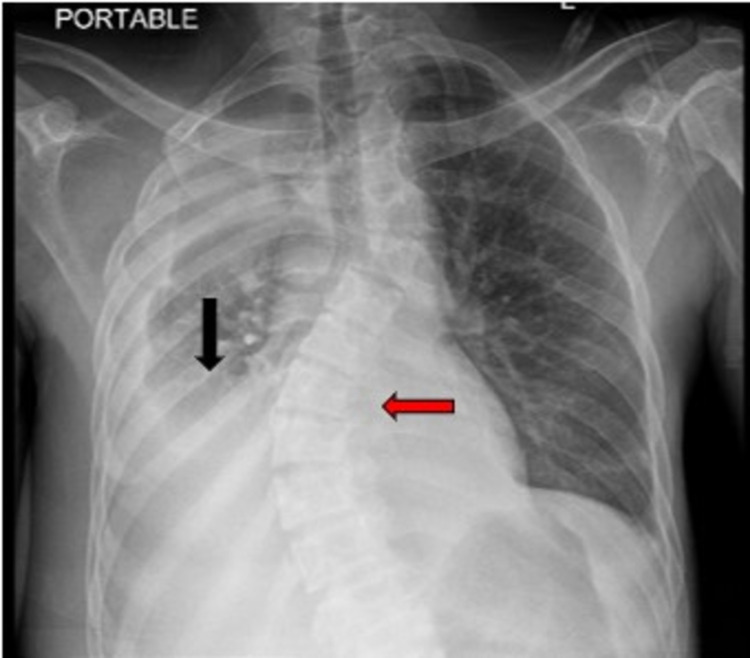
X-ray showing pleural effusions secondary to pneumonia (black arrow), in addition to chronic stable, S-shaped scoliosis (red arrow).

After admission, a chest X-ray was performed and was negative for any acute findings. However, the patient has a history of pleural effusions secondary to pneumonia 6 months prior to this current admission, in addition to a chronic stable, S-shaped scoliosis (Figure 3). 

A metabolic panel, a complete blood count (CBC) hemogram, and cell morphology labs were also ordered after admission (Tables 1, 2). Total immunoglobulin E levels during this admission were measured to be 776 IU/mL, whereas the normal range should be below 144 IU/mL; a chronic low mean corpuscular hemoglobin concentration (MCHC) anemia and an elevated white blood cell count were also significant laboratory findings. A transthoracic echocardiogram previously performed revealed an ejection fraction of 50-55%, with a moderate mitral valve regurgitation leading to a moderately dilated left atrium. A bone aspirate smear and biopsy were done previously showed that in all the cells analyzed normal male chromosome complement was observed, without any evidence of any chromosomal abnormality.

**Table 1:** Lab values on admission of the patient after initial presentation. WBC: white blood cell count; RBC: red blood cell count; HCT: hematocrit; HGB: hemoglobin; MPV: mean platelet volume; MCV: mean corpuscular volume; fL: femtoliter; g/dL: grams per deciliter; mcL: microliter; MCH: mean corpuscular hemoglobin; MCHC: mean corpuscular hemoglobin concentration; RDW: red cell distribution width. The symbols “˄” and “˅ “indicates the value above and below the standard normal ranges, respectively. The reference parameters were determined for approximately 1500 to 2000 individuals ages 20 to 80 (varies slightly by category).

Test	Value	Reference Value
WBC	11.9˄	3.8-10.4 (× 103/microL)
RBC	3.64˅	Male: 4.2-5.7; Female: 3.8-5.0 (× 106/microL)
HGB	8.0 ˅	Male: 13.6-16.9; Female: 11.9-14.8 (g/dL)
HCT	26.0 ˅	Male: 40-50%; Female: 35-43%
MCV	71.4 ˅	82.5-98 (fL)
MCH	21.9 ˅	27-33 (pg)
MCHC	30.7	32-35.2
RDW	18.0 ˄	11.4-13.5%
MPV	9.0	7-12 fL
Platelets	336	Male: 152-324; Female: 153-361 (× 103/microL)
Neutrophils Auto	77.2 ˄	40-60%
Lymphocytes Auto	13.9 ˅	20-40%
Monocytes Auto	7.8	2-8%
Eosinophils Auto	0.8	1-4%
Basophils Auto	0.3	0.5-1%
Neutrophils Absolute	9.20 ˄	1.7-7.0 x 109/L
Lymphocytes Absolute	1.70	1.0-4.8 x 10^9^/L
Monocytes Absolute	0.90	0.30-0.90 x 109/L
Eosinophils Absolute	0.1	0.05-0.50 x 109/L
Basophils Absolute	0.00	0.00-0.30 x 109/L
Hypochromia	2+	
Microcytic	1+	
Anisocytosis	1+	
Large Platelets	Present	

**Table 2:** Lab values for the patient on initial presentation in addition to the last known values for immunoglobulins and other pertinent lab tests. ** These lab values were taken during a hospital admission in December 2020, not during the current admission. AST: aspartate aminotransferase; ALT: alanine aminotransferase; CO2: carbon dioxide; mg/dL: milligrams per deciliter; mEq/L: milliequivalents per liter; g/dL: grams per deciliter; IU/L: International units per liter; mcL: microliter; nmol/L: nanomoles per liter; μmol/mmol: micromole per millimole; kU/L: kilounits per liter. The symbols “˄” and “˅ “indicates the value above and below the standard normal ranges, respectively. The reference parameters were determined for approximately 1500 to 2000 individuals ages 20 to 80 (varies slightly by category).

Test	Value	Reference Value
Glucose	82	80-120 mg/dL
Blood Urea Nitrogen	12.9	6 – 20 mg/dL
Creatinine	0.62 ˅	0.6 – 1.3 mg/dL
Sodium	137	135-147 mEq/L
Potassium	4.5	3.5-5.5 mEq/L
Chloride	101	98-106 mEq/L
CO2	25	23-29 mEq/L
Calcium	9.2	8.5 to 10.2 mg/dL
Anion Gap	11.00	3-10 mEq/L
Phosphorus	3.6	2.5-4.5 mg/dL
Protein, Total	7.4	6.3-8.0 g/dL
Albumin	3.4 ˅	3.5-5.5 g/dL
Bilirubin, Total	0.5	<1.0 mg/dL
ALT	<10 ˅	10-56 IU/L
AST	12	10-40 IU/L
Alkaline Phosphatase	89.7	44 to 147 IU/L
Magnesium	1.9	1.5-3.0 mEq/L
Albumin/ Globulin Ratio	0.9	1.1-2.5
Methylmalonic Acid**	4.33 ˄	< 3.6 μmol/mmol
Vit B1, Whole Blood**	112.8	74-222 nmol/L
C-Reactive Protein, High Sensitivity**	2.190	0.5 to 10 mg/L
Procalcitonin**	0.22 ˄	<0.1 ng/mL
IgG**	1,192	767-1,590 mg/dL
IgE**	776 ˄	1.5-144 kU/L

**Figure 4:**
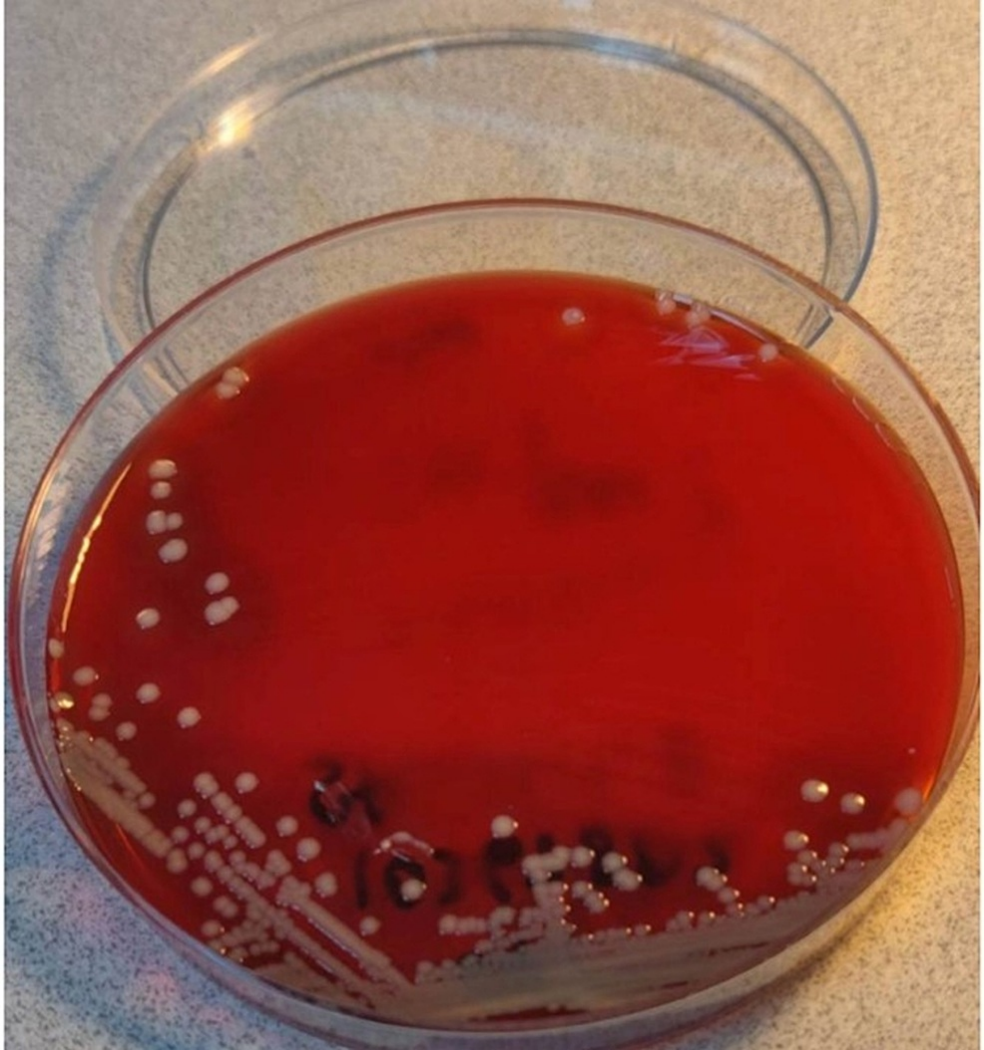
Culture of the coagulase-negative Staphylococcus species cultured from the right axillary abscess.

The wound culture for the current right axillary abscess was positive for moderate coagulase-negative *Staphylococcus* species (Figure 4). A diagnosis of hidradenitis suppurativa of the right axilla secondary to his primary immune deficiency was established and treated with surgical wound debridement with antibiotics and daily wound care.

**Figure 5:**
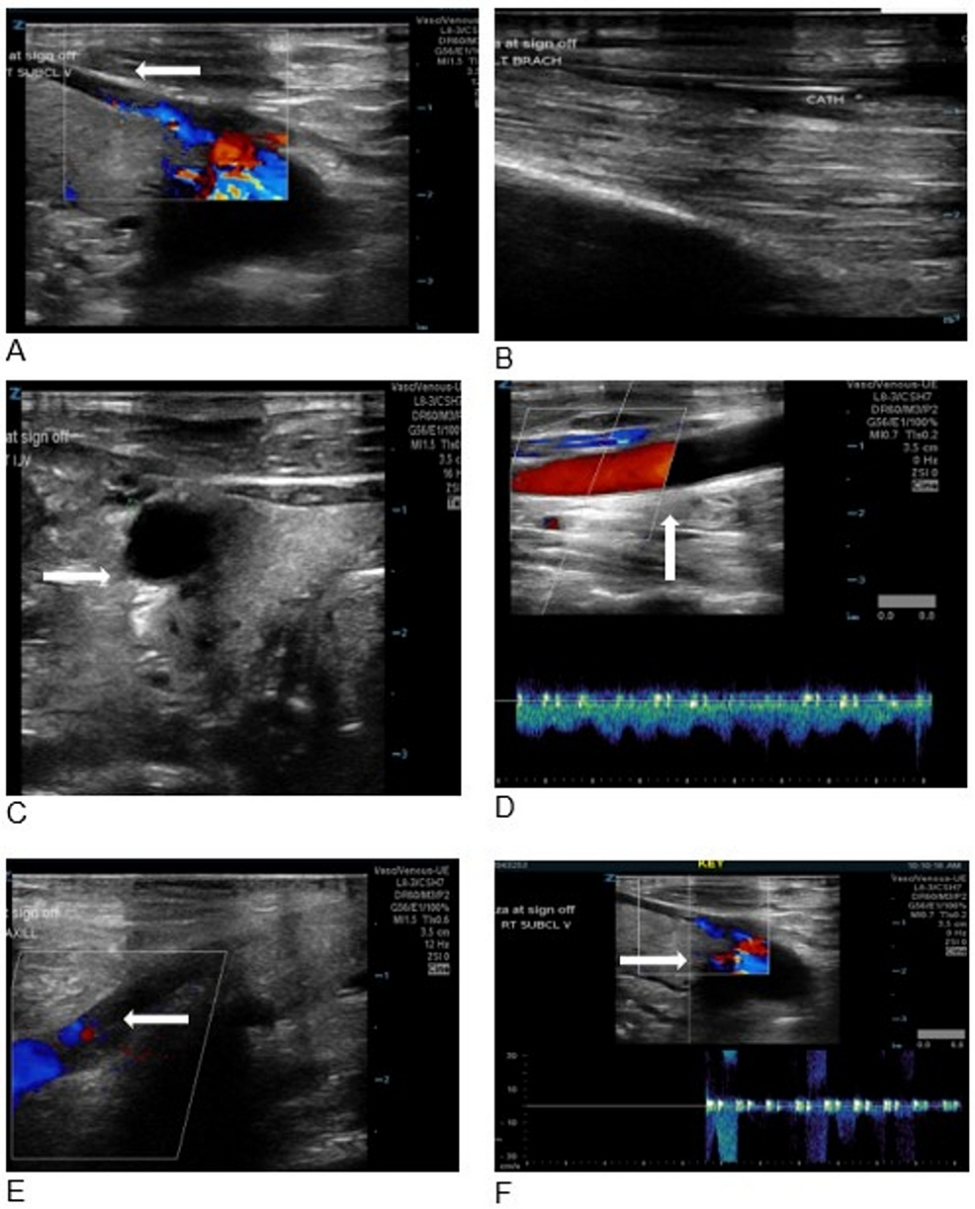
Duplex imaging showing different views of a chronic deep vein thrombosis of right upper extremity (arrows).

The patient also has a history of deep vein thrombosis (DVT) in the upper limb. Duplex imaging of the right upper extremity performed in the last year showed a right-sided chronic DVT with recanalization (Figure 5), without any evidence of left-sided upper extremity DVT.

## Discussion

Hyper-IgE syndrome is a complex and rare primary immunodeficiency disorder classically characterized by a triad of eosinophilia, atopic dermatitis, recurrent skin, and pulmonary infections [1]. However, it has been known to include the anomalies of skeletal, connective tissues, heart, and brain [2,3]. 

HIES has been classified into two types: a autosomal dominant form which has been studied to be a result of STAT3 mutations, which cause an escalation in the production of IgE by B lymphocytes, and a recessive form which is associated with mutations and deletions in DOCK8 and TYK2 genes [4]. The former presents as severe recurrent cutaneous and pulmonary infections associated with systemic signs of inflammation and connective tissue and skeletal abnormalities, whereas the latter is associated with severe cutaneous viral infections, asthma, allergies, and risk of malignancy [5]. Since our patient does not have any family history of this disease, we can assume they either have a sporadic STAT3 mutation or they have the autosomal recessive form. Job syndrome lacks a specific diagnostic criterion and diagnosis is made by clinical manifestations and laboratory abnormalities. Laboratory findings include high IgE levels often above 2000IU/L and eosinophilia [6]. Genetic testing has been considered for the autosomal dominant form and a scoring system is used to calculate the probability of STAT3 mutation [7-9]. Genetic testing was performed to confirm the diagnosis in our case. 

Cold abscesses, which are the hallmark of the disease, are most frequently found on the face and the trunk. They appear very early in the disease course and are rapidly superinfected with *Staphylococcus aureus*, leading to weeping, crusty, and follicular lesions.

Pulmonary complications such as scoliosis exist in 63% of cases, such as seen in our patient as noted in Figure 3. Other common pulmonary complications include recurrent pneumonia due to *S. aureus* resulting in lung abscess, bronchiectasis, and pneumatoceles. These are seen in 77% of the cases, however, bronchiectasis and pneumatoceles were absent in our patient [6-9]. Characteristic facial features such as a prominent forehead, deep-set eyes, prognathism, increased inter-alar distance and broad nasal bridge have an incidence of 100% in patients older than 16 years [9]. These distinct coarse facies were also seen in our case [4]. Vascular complications include tortuosity or dilation of blood vessels [10] and this was seen in our patient as well. 

Long-term antibiotics are the mainstay of treatment as they can prevent and treat serious infections, TMP-SMX and amoxicillin-clavulanic acid are used frequently and are known to significantly decrease the incidence of abscesses and pneumonia. These antibiotics have been used on multiple occasions on our patient to help reduce the severity and incidence of skin and lung infections in addition to frequent baths in a diluted bleach solution. Immunomodulators have not been studied well and interferon gamma has failed to show benefit [10]. In summary, control and curative approaches are still lacking hence, there is a dire need for new therapeutic solutions.

## Conclusions

Job syndrome is obviously a debilitating immunologic phenomenon with an eventful clinical course. Our patient developed his first abscess in late childhood with multiple episodes of soft tissue infection lately and was eventually diagnosed at 18 years of age. Autism was a distinctive association along with scoliosis and chronic deep vein thrombosis, making this case engrossing. No definitive treatment protocol has been established to treat this syndrome, however, frequent clinical evaluation, prophylactic topical and oral antibiotics, close observation, and skincare have been encouraged. 
